# Unraveling the Clinical Quandary: Cryptic Tuberculosis or Splenogonadal Malignancy?

**DOI:** 10.7759/cureus.71992

**Published:** 2024-10-21

**Authors:** Rashika M, Nidhi Elizabeth Jacob, Ghanshyam Verma

**Affiliations:** 1 Radiology, Government Medical College Mahasamund, Kharora, IND; 2 Internal Medicine, Government Medical College Mahasamund, Kharora, IND; 3 Radiodiagnosis, Pt. Jawahar Lal Nehru Memorial Medical College, Raipur, IND

**Keywords:** atypical presentation, cryptic tuberculosis, diagnostic challenge, dissemination, histopathology, microbiological culture

## Abstract

Tuberculosis continues to persist as a significant health problem in multiple parts of the world despite global efforts to eradicate it. A high variability in the clinical manifestation hinders the early diagnosis. Atypical presentations like “cryptic tuberculosis” lack classic clinical and radiological features of the disease and can mimic metastatic cancer, posing a diagnostic challenge. Herein, we report a case of a 70-year-old male with complaints of chronic abdominal pain, who was presumed to have malignant disease of the spleen and testis after clinical and radiological assessment. However, the histopathology and microscopy revealed features of tuberculosis, and a culture test confirmed the diagnosis. Hence, clinicians should be vigilant of the ambiguity of symptoms, especially in immunosuppressed patients and among residents of endemic areas. This can target aggressive efforts to diagnose and treat such unusual presentations of tuberculosis, avoiding unwanted mortality.

## Introduction

Tuberculosis, caused by bacteria of the *Mycobacterium tuberculosis* complex, is one of the deadliest diseases in the world. It is the second leading cause of infectious deaths worldwide after COVID-19. It is widely considered a public health issue in low and middle-income countries [1]. Globally, around 10 million people contract the disease, with over a million deaths every year. A quarter of the global TB cases are reported in India, with an estimated incidence of 2.77 million in 2022 [2]. *Mycobacterium tuberculosis* complex predominantly affects the pulmonary tissue. One-third of the cases can be extrapulmonary, commonly involving lymph nodes, followed by the pleura, genitourinary system, bones and joints, meninges, peritoneum, pericardium, gastrointestinal tract, and other solid organs. However, any organ system can be involved [3].

Disseminated tuberculosis is a mycobacterial infection in two or more non-contiguous sites resulting from lymphohematogenous spread. The term "cryptic tuberculosis" describes patients with less typical findings in chest radiographs who present with disseminated tuberculosis. Lack of localizing signs, normal chest radiography, absence of choroidal tubercles, and negative tuberculin skin test add to the dilemma in diagnosing such forms of tuberculosis [4]. Variability in the clinical picture and resemblance to many other diseases delay the diagnosis of cryptic tuberculosis.

Herein, we present a case of cryptic tuberculosis with splenic and testicular involvement mimicking malignancy.

## Case presentation

A 70-year-old male presented to the medicine OPD with complaints of upper abdominal pain for the past 15 days. There was no associated history of abdominal distention, nausea, or vomiting. Furthermore, there was no fever, weight loss, loss of appetite, night sweats, and cough. The patient had no history of alcoholism and smoking habits. There was no contact history of tuberculosis in the family. On physical examination, he was afebrile, with a heart rate of 84 bpm and blood pressure of 112/86 mmHg. An abdomen examination revealed a soft, non-tender, non-rigid abdomen with grade 2 splenomegaly and hard, non-tender left testis. The right testis was unremarkable. No obvious swelling or sinus was noted in the scrotum. A few enlarged left-sided inguinal lymph nodes were noted. Respiratory, cardiovascular, and neurological system examinations were unremarkable. Baseline blood investigation revealed anemia, leucocytosis, and elevated erythrocyte sedimentation rate. Liver and renal function tests were normal. Tumor markers such as lactate dehydrogenase, alpha-fetoprotein, and beta-human chorionic gonadotropin were found to be insignificant (Table 1).

**Table 1:** Blood investigations.

Parameter	Result	Reference range
Hemoglobin (g/dL)	9	13.5-17.5
Total leucocyte count (per mcL)	17000	4500-11000
Platelet count (per mcL)	200000	150000-450000
Erythrocyte sedimentation rate (mm/hr)	45	<17
Total bilirubin (g/dL)	0.6	0.1-1.2
Aspartate transaminase (IU/L)	15	<40
Alanine transaminase (IU/L)	18	<40
Serum urea (g/dL)	10	5-20
Serum creatinine (g/dL)	0.9	0.6-1.2
Alpha-fetoprotein (µg/L)	3	<10
Lactate dehydrogenase (U/L)	110	<250
Beta human chorionic gonadotropin (IU/L)	1	<5

**Figure 1:**
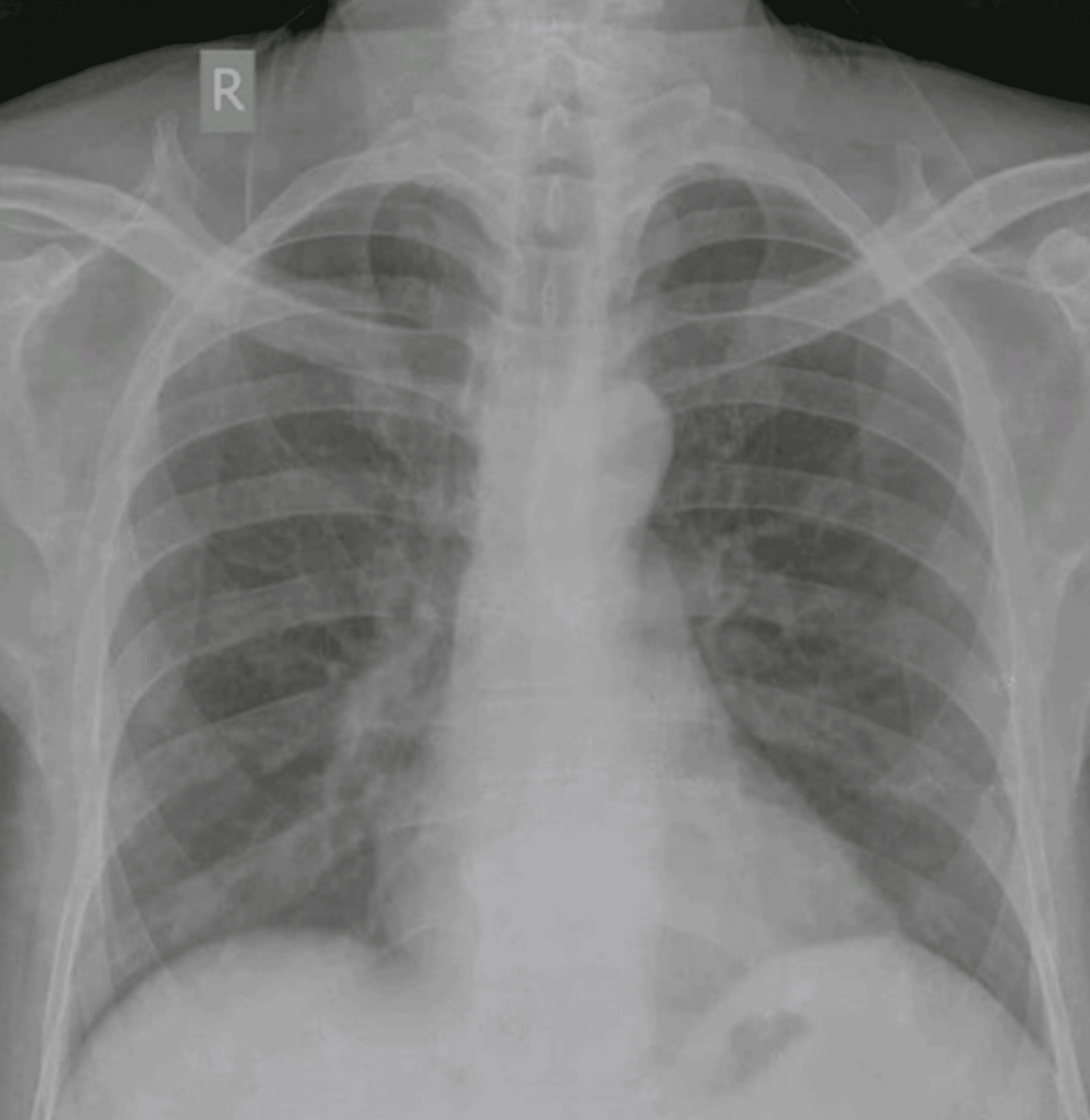
Chest X-ray (posteroanterior view) showed bilateral clear lung fields with normal cardiac shadow.

Serology for HIV, hepatitis B and C, and venereal disease research laboratory (VDRL) were normal. His chest X-ray revealed bilateral clear lung fields with normal cardiac shadow (Figure 1).

**Figure 2:**
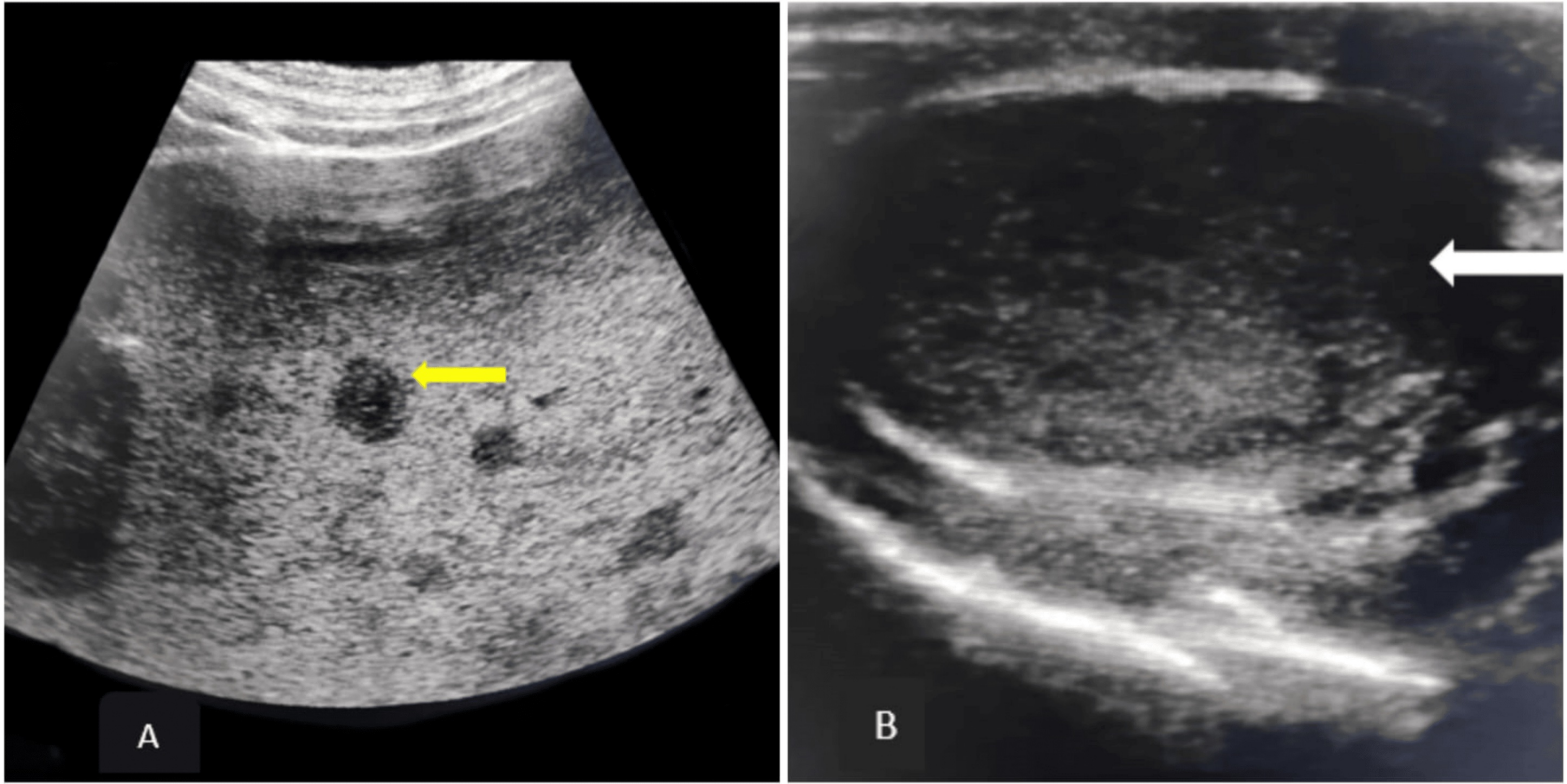
(A) USG of the abdomen showed splenomegaly with multiple variable-sized hypoechoic lesions (yellow arrow) with no obvious internal vascularity. (B) USG of the scrotum showed homogenously hypoechoic left testis (white arrow) with no obvious increase in vascularity.

Abdominal sonography revealed moderate splenomegaly with multiple variable-sized hypoechoic lesions with no obvious internal vascularity, and few of them showed central necrosis. Scrotal sonography revealed a homogenously hypoechoic left testis with no obvious increase in vascularity (Figure 2).

**Figure 3:**
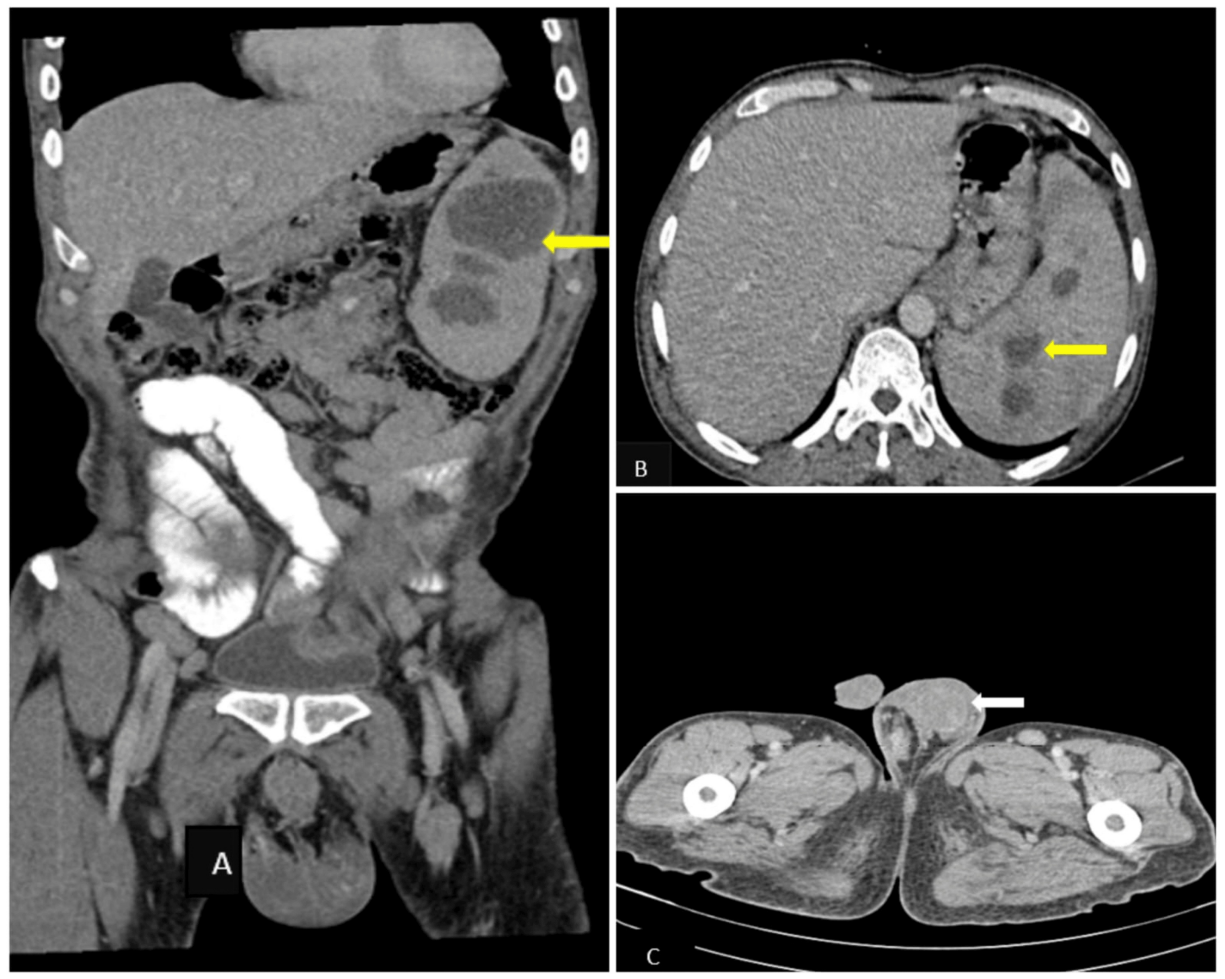
Contrast-enhanced CT of the abdomen in coronal view (A) and axial view (B) showing multiple variable-sized hypodense lesions in the spleen with peripheral rim enhancement (yellow arrow). (C) Contrast-enhanced CT of the pelvis showing heterogeneously enhancing left testis (white arrow) with left inguinal lymphadenopathy.

On contrast-enhanced computed tomography, moderate splenomegaly with multiple variable-sized hypodense lesions in the spleen showed peripheral rim enhancement and heterogeneously enhancing left testis with left inguinal lymphadenopathy. Hence, the probable diagnosis of testicular neoplasm with splenic metastasis was made (Figure 3).

**Figure 4:**
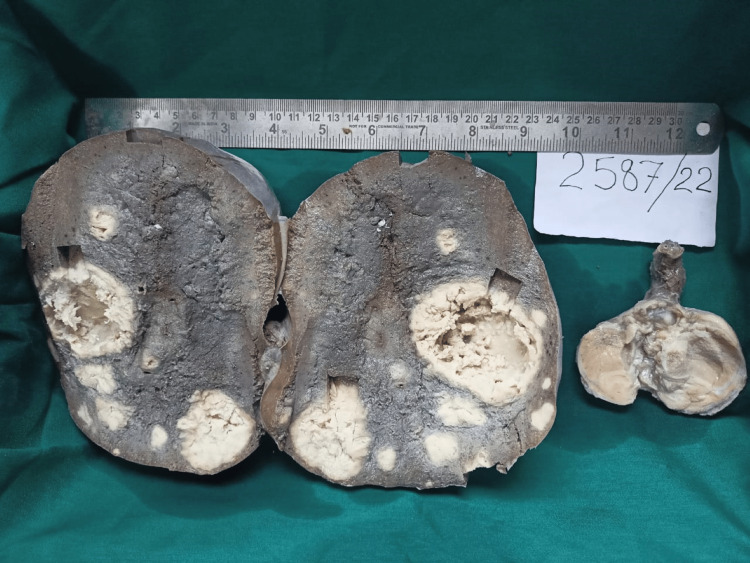
Gross specimen showing few greyish-white solid lesions in the spleen with cheesy white material on the central cut area and solid greyish white lesion on left testis.

Fine needle aspiration from the splenic lesion was attempted and was inconclusive. Hence, the patient was planned for scrotal exploration with high inguinal orchidectomy and splenectomy. Intraoperatively, whitish aspirate from the largest splenic lesion was sent for culture and sensitivity testing. The gross specimen of the spleen showed a few greyish-white solid lesions with yellowish cheesy material on the central cut area. The specimen of the testis showed a solid, greyish-white lesion (Figure 4).

**Figure 5:**
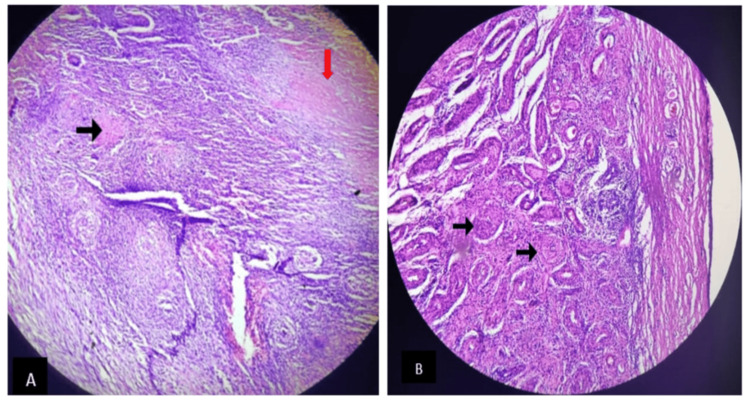
On histopathology, spleen (A) showed multiple granulomas (black arrow) with caseous necrosis (red arrow), and testis (B) showed atrophied and fibrous seminiferous tubules along with multiple granulomas (black arrow).

**Figure 6:**
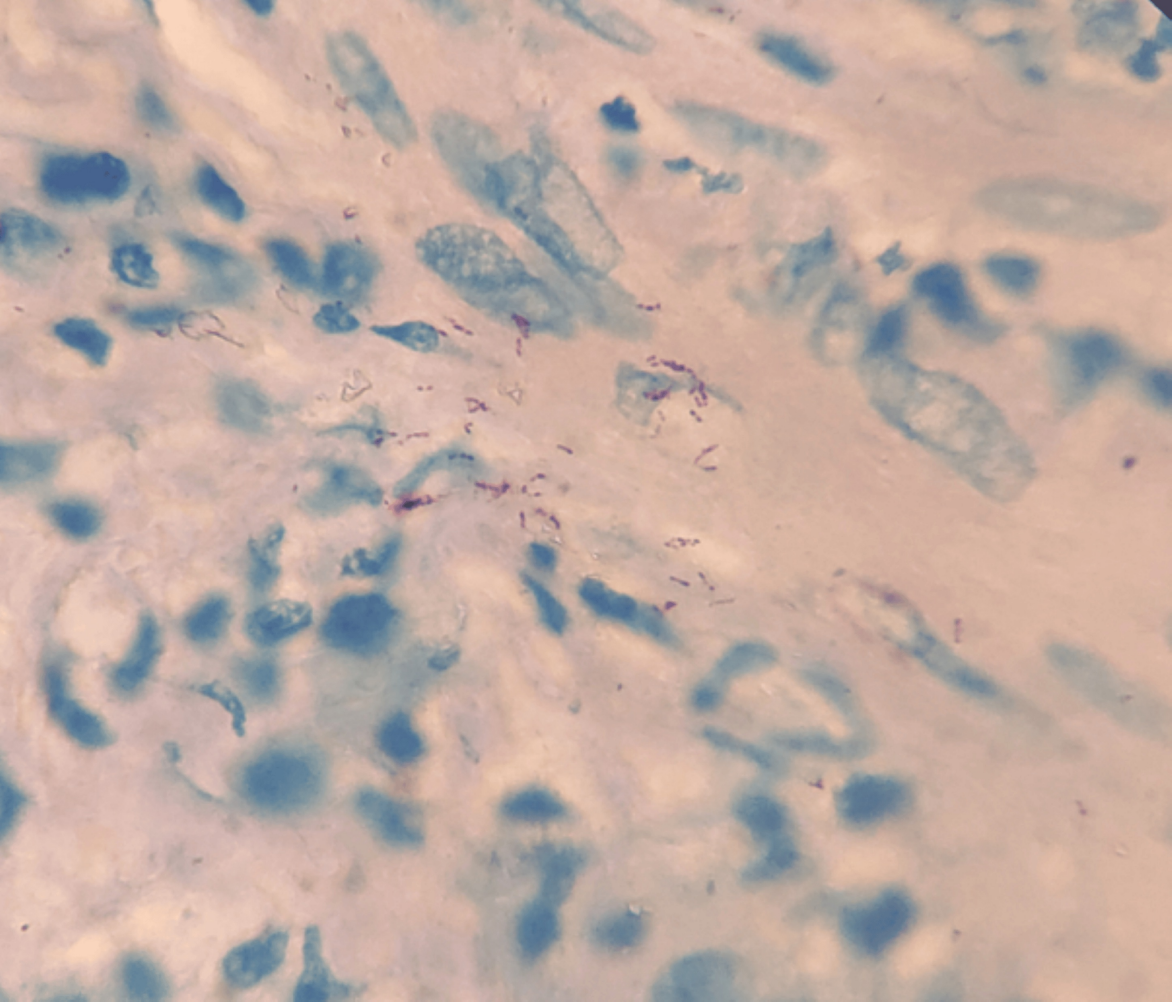
Ziehl-Neelsen (ZN) staining revealed acid-fast bacilli.

On histopathological examination, the spleen showed multiple granulomas with caseous necrosis, and the testis showed atrophied and fibrous seminiferous tubules along with multiple granulomas (Figure 5). Ziehl-Neelsen (ZN) smear microscopy confirmed acid-fast bacilli (Figure 6).

Culture and sensitivity testing revealed *Mycobacterium tuberculosis* growth and showed no resistance to any first-line drug of antitubercular treatment. Hence, the definitive diagnosis of cryptic tuberculosis was made. The patient was started on antitubercular treatment for six months with an intensive phase of two months (isoniazid, rifampicin, ethambutol, and pyrazinamide) and a continuation phase of four months (isoniazid and rifampicin, ethambutol). The follow-up was uneventful.

## Discussion

Dissemination of tuberculosis can either be a sequel of progressive primary infection or reactivation of subclinical infection with subsequent lymphohematogenous spread [5]. It is presumed that tuberculosis, primarily in the lung, causes alveolar epithelial cell erosion, spreading the infection to the left pulmonary vein, the left heart, and finally to the systemic circulation. The bacilli can also breach the alveolar cells to enter lymph nodes and, through lymphatics, enter the systemic venous circulation [6].

Advanced age and immunocompromised status mainly predispose to the dissemination of tuberculosis. This condition is frequently seen in endemic areas. Clinical features of disseminated tuberculosis can be diverse depending on the organ involved. In the elderly, typical features like fever may be absent, and the patient may present with emaciation, simulating metastatic carcinoma [7].

On rare occasions, disseminated tuberculosis presents with a normal or non-miliary pattern in chest radiographs. This form of the disease is labeled as “cryptic tuberculosis,” making the primary diagnosis difficult and causing mismanagement of the patient. It can also go undiagnosed until autopsy. The diagnosis of cryptic tuberculosis requires culture positivity from two or more non-contiguous organs or culture positivity from an organ, along with a histopathological demonstration of caseating granuloma from any other non-contiguous organ [8].

In our case, the detailed clinical assessment and extensive investigations eventually led to the diagnosis of splenic and testicular tuberculosis. Splenic tuberculosis is usually a part of disseminated disease. Isolated cases have been rarely reported. Sometimes, it is associated with spontaneous splenic rupture or hypersplenism [9]. On imaging, it can present as isolated splenomegaly, micronodular, or macronodular form. The macronodular disease can have solitary or multifocal lesions. On USG, well-defined hypoechoic lesions can be seen, with or without central necrosis. On CT, a hypodense lesion with peripheral rim enhancement can be seen. The differential diagnosis of splenic tuberculosis includes leukemia, lymphoma, metastases, sarcoidosis, hemangioma, cyst, and fungal infection [10,11].

In the case of genital tuberculosis, epididymis is primarily involved. The testicles are usually affected in contiguity with the epididymis, as the blood-testicle barrier plays a protective role [12]. However, some reported cases of isolated testicular tuberculosis have been seen, as in our case. Tubercular epididymo-orchitis or isolated orchitis can mimic other inflammatory processes, torsion, or testicular tumors. On USG, the testis appears homogenously or heterogeneously enlarged with hypoechoic echotexture, with or without nodularity. Sometimes multiple hypoechoic nodules can be seen [13,14].

There is no consensus on the diagnostic workup of cryptic tuberculosis. The ultimate aim is to identify the affected site to obtain appropriate specimens for AFB smear, polymerase chain reaction (PCR), mycobacterial culture, and histopathological examination [8,15]. Nowadays, cross-sectional imaging with ultrasound (US), multidetector computed tomography (MDCT), and magnetic resonance imaging (MRI) plays an important role in the identification of affected non-contiguous organs. PET-CT has recently been used in disseminated disease as 18F-fluorodeoxyglucose can also accumulate in inflammatory cells similar to malignant cells [16]. Though multiple modalities are available for diagnosing visceral tuberculosis, a definitive diagnosis can be made by histopathological examination and mycobacterial culture [17].

## Conclusions

The health targets of the United Nations Sustainable Development Goals include ending the tuberculosis epidemic by 2030. However, tuberculosis continues to persist as a major global health threat. Presentations with varying and nonspecific signs and symptoms make diagnosing this mycobacterial infection a formidable task. With the advent of imaging modalities, previously undiagnosed cases of cryptic tuberculosis have been increasingly identified. However, a close resemblance to multiple unrelated conditions, even in radiology, delays the prompt diagnosis and treatment, as in our case. Keeping this in mind, adequate knowledge and a high index of suspicion are imperative for the timely recognition and management of this public health crisis.
